# Plant miRNAs found in human circulating system provide evidences of cross kingdom RNAi

**DOI:** 10.1186/s12864-017-3502-3

**Published:** 2017-03-14

**Authors:** Yu-Chen Liu, Wen Liang Chen, Wei-Hsiang Kung, Hsien-Da Huang

**Affiliations:** 1 Institute of Bioinformatics and Systems Biology, National Chiao Tung University, HsinChu, Taiwan; 2 Department of Biological Science and Technology, National Chiao Tung University, HsinChu, Taiwan; 3 Center for Bioinformatics Research, National Chiao Tung University, Hsinchu, 300 Taiwan; 4 Department of Biomedical Science and Environmental Biology, Kaohsiung Medical University, Kaohsiung, 300 Taiwan

**Keywords:** Circulating miRNAs, Plant miRNAs, miR2910, Cross Kingdom RNAi

## Abstract

**Background:**

Emerging evidence indicates that plant miRNAs can present within human circulating system through dietary intake and regulate human gene expression. Hence we deduced that comestible plants miRNAs can be identified in the public available small RNA sequencing data sets.

**Results:**

In this study, we identified abundant plant miRNAs sequences from 410 human plasma small RNA sequencing data sets. One particular plant miRNA miR2910, conserved in fruits and vegetables, was found to present in high relative amount in the plasma samples. This miRNA, with same 6mer and 7mer-A1 target seed sequences as hsa-miR-4259 and hsa-miR-4715-5p, was predicted to target human JAK-STAT signaling pathway gene SPRY4 and transcription regulation genes.

**Conclusions:**

Through analysis of public available plasma small RNA sequencing data, we found the supporting evidence for the plant miRNAs cross kingdom RNAi within human circulating system.

**Electronic supplementary material:**

The online version of this article (doi:10.1186/s12864-017-3502-3) contains supplementary material, which is available to authorized users.

## Background

Micro RNAs (miRNA) are about 22 nucleotides single strand non-coding RNAs, which regulate protein coding genes expression through guiding protein-RNA complex toward mRNAs [1, 2]. Given the conventional recognition that expression of most of the genes are regulated by miRNAs in mammal, research of miRNAs’ roles in pathogenesis in diseases and biological processes is foreseeable [3]. In recent years, stable cell free miRNAs had been found in human circulating system derived samples such as plasma, urine and saliva [4]. Circulating miRNAs are studied to be used as diagnosis biomarkers in many diseases [5].

Plant miRNA miR168a was found to present in the sera of mammals including human and regulate human gene LDLRAP1 [6]. Positive and negative results of experimental test on whether exogenous miRNAs can present in animal circulating system through food ingestion has been published in the following years [7–10]. Until recently, another indicates that plant miRNAs can present within human circulating system through dietary intake and regulate human gene expression emerged. Broccoli sourced miR159 was found in human sera and proved to inhibit breast cancer growth through targeting the gene TCF7 [11].

Given the assumption that certain comestible plants miRNAs can be absorbed through ingestion and accumulate within human sera, we collected and examined 410 public available small RNA sequencing data sets, detected the existence of plant miRNAs through comparing to genomes of 5 different plant model organism *Arabidopsis thaliana*, *Triticum aestivum*, *Oryza sativa*, *Zea mays*, and *Brachypodium distachyon*. In results, we identified abundant plant miRNAs sequences from 410 human plasma small RNA sequencing data sets. Amount of these exogenous miRNA was compared to the ubiquitous human miRNA presented in the samples. One particular plant miRNA miR2910, conserved in fruits and vegetables, was found to present in high relative amount in the plasma samples. This miRNA, with same 6mer and 7mer-A1 target seed sequence as hsa-miR-4259 and hsa-miR-4715-5p, was predicted to target human JAK-STAT signaling pathway gene SPRY4 and transcription regulation genes through miRTar [12].

This discovery not only provides new evidence of cross kingdom RNAi between human and plant, but also expanded the potential of dietary miRNA treatment on a wide range of variable diseases.

## Results and discussion

A total 1,301 plant miRNAs, including 654 homologs, with at least one read presented in the sample were identified from the selected transcriptome sequencing runs. Count of the reads within each sample is available in (Additional file 1: Table S1). The relative abundance of these miRNAs were compared to detected human miRNAs. Among them we found a singular case, peu-MIR2910, can be found ubiquitously within the selected samples. In some samples, up to more than one thousand copy of peu-MIR2910 can be found, which is more abundant than most of the human miRNAs detected within the samples. Beside peu-MIR2910, up to hundreds of copies of peu-MIR2916 in 379 samples, peu-MIR2914 in 359 samples, and tae-MIR2018 in 353 samples were also found.

### The detected plant miRNAs are not human originated or sample contamination

Sequence alignment of the detected plant miRNAs sequence onto human genome was performed with BLAST [13] and resulted in zero alignment hit. Alignment of the conventional adaptor sequences to the plant miRNAs also resulted with zero hits. Through this result we are convinced that these detected plant miRNA sequence were not originated from conserved human sequences or adaptors contamination.

### The plant miRNA peu-MIR2910 is conserved within comestible plants

Among the 1,301 plant miRNAs detected in our collected samples, miR2910 presented in all the 404 examined runs in relatively high abundance. Assuming that the source of plant miRNAs present in human plasma originated from food ingestion, then miR2910 must be evolutionally conserved in a wide variable kinds of comestible plants.

From the reports of recent years we found that peu-MIR2910 was reported to be expressed within one of the model organism we used, *Zea mays* [14]. As one of the main grain consumed all over the world, maize can potentially the source of the miR2910 detected in the data sets. On the other hand, we also found that miR2910 is conserved within fruits and vegetables. Melon [15], Sorghum [16], tomato [17], tea [18] and oil palm [19] are also the potential source of miR2910. All these evidences support the assumption that the peu-MIR2910 present in the human plasma samples originate from food ingestion. On the other hand, peu-MIR2914 and peu-MIR2916 were also found conserved within oil palm [20]. Conserved tae-MIR2018 was found in wheat [21, 22].

### Plant miR159 and miR168a were also detected in the examined samples

To verify the analysis result with previous reports, we examined the amount of detected miR159 and miR168a within the examined samples. As listed in the (Additional file 1: Table S1), despite presenting in relatively small amount (less than 4 copies), miR159 can be found in 32 samples while miR168a can be found in 7 samples. The difference might be result from the difference of the diet habit. Most of the examined samples were presumably collected from Wisconsin, USA [23]. The dietary habit of the subjects can be much different from the subjects collect in China [6] and California [11]. On the other hand, given the fact that only roughly 1% of all the adaptor trimmed reads can be aligned onto plant genomes, as well as the generally low concentration of miRNA within plasma samples, detection of the miR159 as well as miR168a in less than 4 copy within one sample can still be considered as supporting evidence of the two previous reports.

### Human genes can potentially be targeted by peu-MIR2910 and other plant miRNAs

In the previous reports [6, 11], plant miRNAs present in human circulating system were proven to be able to target human genes through similar AGO protein correlated mechanism. Given the assumption that miRNAs with similar or same target seed sequences can target same sets of genes, we tested the similarity of plant miRNAs and human miRNAs in target seed regions.

**Fig. 1:**
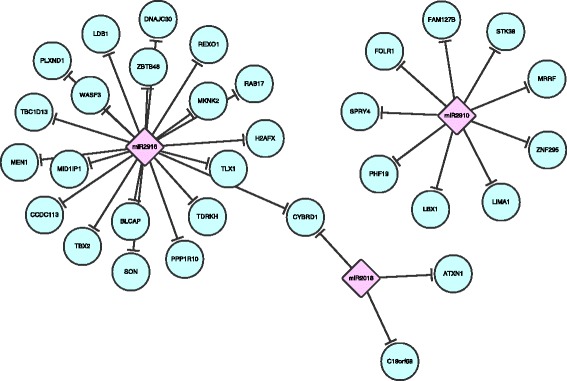
Predicted target human genes of abundant plant miRNAs. The predicted miR2910, miR2916 and miR2018 targets through miRTar [12] are illustrated in this figure. The pink diamond shape nodes represent plant miRNAs while the round blue nodes represent human genes

Through the result of the target seed analysis, we found that despite the whole sequence, including the hairpin, of peu-MIR2910 cannot be aligned onto human genome, the 6mer and 7mer-A1 target seed sequences of miR2910, CCAACT and CCAACTA, is the same as hsa-miR-4259 and hsa-miR-4715-5p. Given the previously reported case of miR168a [6] and miR159 [11], we hypothesis that peu-MIR2910 within the circulating system can target human genes as well. From the 51 target genes of hsa-miR-4259 and hsa-miR-4715-5p reported in miRTarBase [24], and the mature sequence of miR2910, we found 10 possible target genes predicted through miRTar [12], as illustrated in Fig. 1. We found that miR2910 can potentially target on 5′ UTR of LIMA1, and the CDS of CTNND1, FOLR1, LBX1 STK38, FAM127B, PHF19, ZNF295, SPRY4 and MRRF.

Interestingly, we also found that miR2916 has exact same 8mer target seed sequence, AGTCCCCA, with human miRNA hsa-miR-4652-5p. From the 40 target genes of hsa-miR-4652-5p reported in miRTarBase [24], and the mature sequence of miR2916, we found other 20 possible target genes predicted through miRTar [12]. Same analysis process was conducted on miR2018, and three potential human gene targets were found. These findings were also illustrated in Fig. 1.

From the gene sets enrichment, we found that LBX1, PHF19, STK36, ZNF295 and CTNND1 are associated with regulation of transcription, while SPRY 4 belongs to human JAK-STAT signaling pathway, as summarized in Table 1. CTNND1 is also associated with Adherens junctions and endocytosis [25]. We also found that three genes MEN1, LDB1 and TLX1, predicted to be targeted by miR2916 were associated with Transcriptional misregulation in cancer, as summarized in Table 2.

**Table 1:** Gene set enrichment of miR2910 target genes

Category	Term	*P* Value	Genes
GOTERM_CC_FAT	GO:0042995 ~ cell projection	0.026595	FOLR1, CTNND1, SPRY4
SP_PIR_KEYWORDS	developmental protein	0.048812	LBX1, STK36, SPRY4
GOTERM_BP_FAT	GO:0045449 ~ regulation of transcription	0.049408	LBX1, PHF19, STK36, ZNF295, CTNND1
INTERPRO	IPR011989: Armadillo-like helical	0.059853	STK36, CTNND1
SP_PIR_KEYWORDS	transcription regulation	0.060318	LBX1, PHF19, ZNF295, CTNND1
SP_PIR_KEYWORDS	Transcription	0.06372	LBX1, PHF19, ZNF295, CTNND1

This table summarized the gene set enrichment of miR2910 targets. The human genes listed here were predicted to be targeted by plant miR2910 through sequence analysis. The terms were originated from the web based tool DAVID [33]

**Table 2:** Gene set enrichment of miR2916 target genes

Category	Term	*P* Value	Genes
KEGG_PATHWAY	ptr05202: Transcriptional misregulation in cancer	0.011556	MEN1, LDB1, TLX1
GOTERM_BP_DIRECT	GO:0043484 ~ regulation of RNA splicing	0.02339	SON
INTERPRO	IPR000467:G-patch domain	0.025805	SON
UP_KEYWORDS	DNA-binding	0.028196	TBX2, PPP1R10, H2AFX, TLX1
INTERPRO	IPR014720: Double-stranded RNA-binding-like domain	0.029124	SON
GOTERM_CC_DIRECT	GO:0071011 ~ precatalytic spliceosome	0.033471	SON
SMART	SM00443:G_patch	0.036062	SON
GOTERM_BP_DIRECT	GO:0000910 ~ cytokinesis	0.036161	SON
GOTERM_MF_DIRECT	GO:0003723 ~ RNA binding	0.037823	SON, TDRKH
GOTERM_CC_DIRECT	GO:0000781 ~ chromosome, telomeric region	0.046328	PPP1R10, H2AFX
GOTERM_BP_DIRECT	GO:0006397 ~ mRNA processing	0.053776	SON
GOTERM_BP_DIRECT	GO:0000226 ~ microtubule cytoskeleton organization	0.058753	SON
GOTERM_BP_DIRECT	GO:0032092 ~ positive regulation of protein binding	0.063706	MEN1, PLXND1
GOTERM_CC_DIRECT	GO:0000785 ~ chromatin	0.073822	MEN1, PPP1R10
UP_KEYWORDS	Nucleus	0.081889	TBX2, PPP1R10, H2AFX, TLX1
GOTERM_CC_DIRECT	GO:0005634 ~ nucleus	0.08342	MEN1, TBX2, LDB1, MKNK2, H2AFX, MID1IP1, TLX1
GOTERM_BP_DIRECT	GO:0051726 ~ regulation of cell cycle	0.091709	SON

This table summarized the gene set enrichment of miR2916 targets. The human genes listed here were predicted to be targeted by plant miR2916 through sequence analysis. The terms were originated from the web based tool DAVID [33]

## Methods

**Fig. 2:**
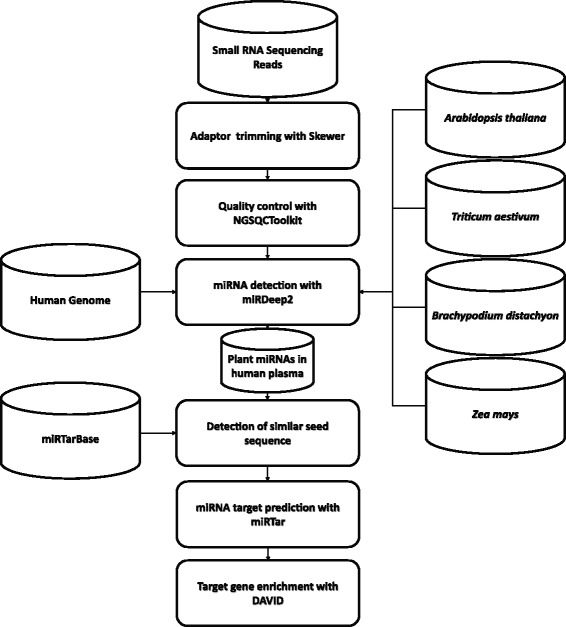
Summary of the data analysis process. The data analysis pipeline designed to detect the comestible plants miRNAs in human plasma samples is summarized in this figure. The collected human circulating small RNA-seq reads were align to the plant genome. The existence of plant miRNAs was examined through the algorithm of miRDeep2 package [29]

The data analysis process in this research is summarized in Fig. 2. Small RNA sequencing data sets of human plasma was dowloaded from NCBI SRA [26]. The adaptors within the data set were trimmed with the tool Skewer [27]. Quality control of the remaining reads was further conducted through NGSQCToolkit [28]. The miRNA detection was performed through miRDeep 2 [29]. To detect the plant miRNAs within the samples, we collected the genomes of 5 different plant model organism *Arabidopsis thaliana*, *Triticum aestivum*, *Oryza sativa*, *Zea mays*, and *Brachypodium distachyon*. The assembly version used were GCA_000001735.1, GCA_000005505.2, GCA_001305255.1, GCA_000210335.1 and GCA_000210335.1, which were downloaded from NCBI assembly. The plant miRNAs used for detection were collected from the database PMRD [30].

The human plasma small RNA sequencing reads were aligned onto each of the plant genomes through Bowtie [31] and the miRDeep2 package [29]. Whether the plant miRNAs presented in the sample was determined by the copy of read aligned on the genomes, precursor and mature sequences of miRNAs. On the other hand, the HG19 human genome was used to detect the human miRNA. The target seed sequences [3] of the detected plant miRNAs were compared to the human mature miRNA sequence downloaded from miRBase [32]. Target genes of human miRNAs with close target seed sequences were assumed to be potentially targeted by the plant miRNAs. The target gene of these human miRNAs were acquired from miRTarBase [24]. Finally these target genes and the sequence of the interested plants miRNAs were used as candidates for miRNA target prediction. The target prediction was conducted through miRTar [12]. The target gene prediction of miRNAs was conducted on 3′UTR, CDS and 5′UTR of the genes, with the threshold of MFE ≦−14 and score ≧140. The gene set enrichment was performed with DAVID [33].

### Conclusions

Through analysis of public available plasma small RNA sequencing data, we found the supporting evidence for the plant miRNAs cross kingdom RNAi within human circulating system.

Singular amount of plant miRNA peu-MIR2910, conserved in fruits and vegetables, was found in the plasma samples presumably collected from the population of Wisconsin, USA. This miRNA, with same 6mer and 7mer-A1 target seed sequences as hsa-miR-4259 and hsa-miR-4715-5p, CCAACT and CCAACTA, was predicted to target human JAK-STAT signaling pathway gene SPRY4 and transcription regulation genes. Whether the detected miR2910 can be absorbed into circulating system through food ingestion should be further tested through xenograft experiments.

## Additional file

Additional file 1: contains supplemental table 1, which summarize count of the reads of plant miRNAs found within each sample. (CSV 2 mb)
